# DGCR8 Microprocessor Subunit Mutation and Expression Deregulation in Thyroid Lesions

**DOI:** 10.3390/ijms232314812

**Published:** 2022-11-26

**Authors:** Lia Rodrigues, Sule Canberk, Sofia Macedo, Paula Soares, João Vinagre

**Affiliations:** 1 Escola Superior de Saúde do Instituto Politécnico do Porto, Rua Dr. António Bernardino de Almeida, 4200-072 Porto, Portugal; 2 Instituto de Investigação e Inovação em Saúde da Universidade do Porto (I3S), Rua Alfredo Allen, 4200-135 Porto, Portugal; 3 Instituto de Patologia e Imunologia Molecular da Universidade do Porto (Ipatimup), Rua Júlio Amaral de Carvalho, 4200-135 Porto, Portugal; 4 Instituto de Ciências Biomédicas da Universidade do Porto (ICBAS), Rua Jorge Viterbo Ferreira, 4050-313 Porto, Portugal; 5 Faculdade de Medicina da Universidade do Porto, Alameda Prof. Hernâni Monteiro, 4200-319 Porto, Portugal

**Keywords:** miRNA, thyroid cancer, microprocessor complex, DGCR8

## Abstract

DGCR8 emerged recently as miRNAs biogenesis pathway protein with a highlighted role in thyroid disease. This study aimed to characterize this miRNA biogenesis component, in particular the p.(E518K) mutation and DGCR8 expression in a series of thyroid lesions. The series of thyroid lesions was genotyped for the c.1552G>A p.(E518K) mutation. When frozen tissue was available, *DGCR8* mRNA expression was analysed by qPCR. Formalin-fixed paraffin-embedded tissues were studied for DGCR8 immunoexpression. We present for the first time the p.(E518K) mutation in a case of poorly differentiated thyroid carcinoma and present the deregulation of *DGCR8* expression at mRNA level in follicular-patterned tumours. The obtained data solidify DGCR8 as another important player of miRNA-related gene mutations in thyroid tumorigenesis, particularly in follicular-patterned thyroid tumours.

## 1. Introduction

Canonical miRNAs are a class of small (22 nucleotides (nt)), non-coding single stranded RNAs essential for normal development [1,2]. miRNA genes are transcribed by RNA polymerase II (RNA pol II) into long, poly-adenylated, and capped primary miRNAs (pri-miRNAs) in the nucleus [3,4,5]. These structured RNAs are processed by the microprocessor complex—a trimeric nuclear complex composed by two DiGeorge Critical Region 8 (DGCR8) proteins bound to one Drosha Ribonuclease III (DROSHA)—and converted in the precursor miRNAs (pre-miRNAs) [3,5]. The pre-miRNAs are then exported to the cytoplasm by the nuclear transport receptor exportin-5 (XPO5), where the Dicer ribonuclease III (DICER) cleaves the base of the loop to generate about 21 to 24 nt double-strand miRNA duplex [3]. The duplex is unwound, and one strand is preferentially selected to bind to one of the Argonaute (AGO2) proteins to generate the final and mature form of miRNA [5,6]. This mature miRNA is incorporated into a ribonucleoprotein complex, known as the RISC (RNA-Induced Silencing Complex) [1,3,5]. The miRNAs regulate gene expression post-transcriptionally, acting as a negative regulator of gene expression, by guiding the RISC to their cognate sites of target mRNAs [7,8]. The targeted mRNA will be initially subjected either to cleavage or translation repression by inhibiting ribosomal access, depending on whether the miRNA: mRNA pairing is perfect or not, respectively [1,3,8,9].

The miRNAs biogenesis pathway holds a key role in the proper development of the thyroid gland, being miRNAs necessary for accurately establishing thyroid follicles and hormone synthesis [7,10,11]. The miRNAs may affect the initiation, development, and progression of cancer through alteration of the expression levels of their target genes [12]. The normal thyroid gland highly expresses miRNAs that are commonly downregulated in thyroid carcinomas (TC), suggesting a role for specific miRNAs as key factors in the development and progression of TC as they are acting as important tumour-suppressors [2,10,13]. The activation of oncogenes is a known cause of miRNAs global deregulation in thyroid cells, with a marked reduction in suppressive miRNAs and activation of oncogenic miRNAs [10]. Altered miRNA expression in cancer is quite often related to the malfunction of DICER and miRNA-machinery associated proteins [10]. Alongside the alterations in expression, mutations in the genes involved in the processing of miRNAs are reported both at the somatic and germline levels [14]. The discovery of *DICER1* germline mutations identified the first cancer predisposition syndrome that was caused by impaired miRNA biogenesis. As a component of DICER1 syndrome, a series of thyroid disorders were identified, reinforcing the relevance of miRNA in human thyroid function [8,10,15]. Somatic *DICER1* mutations are reported mostly in follicular-patterned lesions of thyroid [14]. Other somatic mutations in genes (*DROSHA*, *DGCR8*, *TARBP2*, *XPO5*) encoding miRNA biogenesis proteins are reported [2,7]. 

DGCR8 is the microprocessor component that directly interacts with pri-miRNAs [6,16]. Knock-down of *DGCR8* results in, as observed upon *DROSHA* deplete on, a pronounced decrease in mature miRNA level affecting the expression of cancer-related genes [3]. The conditional knock-out of *DGCR8* at early stages of thyroid development leads to severe hypothyroidism with almost undetectable free thyroxine, thyroid tissue disorganization and few follicular structures [10,11]. Bartram et al. [11] observed in *DGCR8* knock-out mice severe hypothyroidism which can explain the lethality of loss of *Dgcr8* in the thyroid gland. Impaired miRNA processing caused by the aberrant expression of miRNA biosynthesis genes *DGCR8* and *DROSHA* can noticeably promote tumorigenesis, being correlated with pathophysiology of cancers [17,18]. *DGCR8* gene localizes to chromosome 22 (22q11.2) [7]. The 22q11.2 microdeletion leads to upregulation of several pri-miRNAs accompanied by downregulation of a subset of mature miRNAs, being of major interest when dealing with thyroid defects as is commonly found to be lost in these lesions leading to loss of heterozygosity (LOH) [19]. This 22q deletion is also identified to lead to *DGCR8* haploinsufficiency, resulting in a decrease in microprocessor efficiency and deregulation of miRNA expression [20]. Previous studies identified the mutation c.1552 G>A in exon 6 of *DGCR8* that codes a glutamic acid to lysine substitution in position 518, p.(E518K). This mutation is a somatic hotspot in Wilms’ tumours, it has been identified in two papillary thyroid carcinomas (PTC) and in the germline of three-generation family with euthyroid multinodular goitre (MNG) and schwannomatosis; and, more recently in 2 widely invasive follicular thyroid carcinomas (FTC) [7,21,22]. A biallelic alteration of *DGCR8* was described in all cases: p.(E518K) mutation plus somatic loss of the whole chromosome 22 [7]. This combination suggests a critical role for p.(E518K) in predisposing to tumour development. Somatic loss of chromosome 22, containing the wild-type (WT) allele, appears to be required for tumorigenesis, indicating that *DGCR8* acts as a tumour suppressor gene [7,23]. This mutation disrupts global miRNA production and *DGCR8*-mutated tumours display a specific miRNA profile different from *DGCR8*-WT tumours [7,23]. The globally reduced levels of miRNA could be due to reduced catalytic efficiency or changes in specificity [23]. Vardapour et al. [23] described that subsequent to altered miRNA levels, the expression of mRNA targets was likewise changed. In silico modelling by multiple algorithms predict that p.(E518K) mutation is pathogenic, with a reduction in the affinity of RNA binding to DGCR8 [7]. The p.(E518K) is also predicted to be expressed at the RNA level, does not affect splicing, and is not subject to nonsense-mediated decay [7]. Previous studies described that *DGCR8* p.(E518K) cells display partial proliferation and differentiation defect [23].

The important role of DGCR8 in miRNA processing and its previous association in thyroid gland alterations points him as a very attractive target. In this study, our aim was to characterize DGCR8 microprocessor subunit in a series of thyroid lesions by genotyping *DGCR8* recurrent mutation p.(E518K), to evaluate *DGCR8* mRNA and protein expression by real-time PCR (qPCR) and immunohistochemistry (IHC), respectively.

## 2. Results

### 2.1. c.1552G>A p.(E518K) in a Poorly Differentiated Thyroid Carcinoma

A total of 226 samples from 209 patients were genotyped. The samples included 5 normal thyroid tissues (NT), 15 multinodular goiters (MNG), 86 follicular thyroid adenomas (FTA) (2 cases with normal tissue adjacent to tumour (NTAT), 22 follicular thyroid carcinomas (FTC) (6 cases with NTAT), 80 papillary thyroid carcinomas PTC (2 cases with NTAT), 5 poorly differentiated thyroid carcinomas (PDTC) (1 case with NTAT) and 2 anaplastic thyroid carcinomas (ATC). The total of 80 PTC cases included classical variants of PTC (*n* = 44; cPTC), follicular variants of PTC (*n* = 22; FV-PTC) and other variants of PTC (*n* = 14; OV-PTC: 3 tall cell PTC, 4 oncocytic PTC, 5 diffuse sclerosing PTC and 2 solid trabecular PTC). In Table 1 are represented the clinicopathological and molecular parameters collected regarding the studied cohort. We also evaluated germline DNA (blood) of 12 probands from MNG with familial association. A part of the samples used in this series was previously characterized for mutations in *TERT, BRAF,* and *NRAS* genes and for *RET/PTC* and *PAX8/ PPARg* rearrangements [24].

We identified a *DGCR8* mutation—c.1552G>A, p.(E518K)—in an insular variant of a PDTC, Table 1; to our knowledge the first report of this alteration in this subtype. This somatic mutation was not detected in the corresponding NTAT, excluding it to be a germline alteration. Regarding other genetic events, no rearrangements *RET/PTC* and *PAX8/ PPARg, TERT, NRAS* and *BRAF* mutations were detected in the same case. This mutation occurred in an older patient (82 years-old) with a 10 cm tumour presenting poor prognosis characteristics, such as invasion of capsule and extra-thyroidal invasion. No further mutations were detected, including the 12 probands from MNG with familial association.

### 2.2. Deregulation of DGCR8 mRNA Expression in Follicular-Patterned Tumours

**Figure 1:**
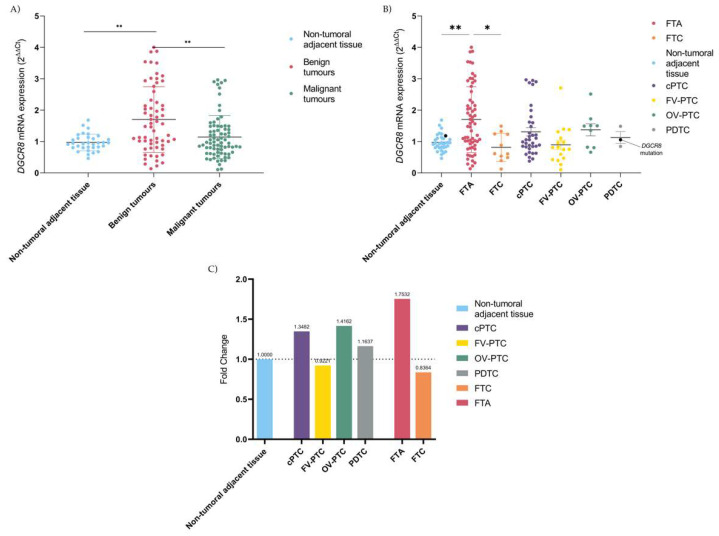
Expression of DGCR8 mRNA in thyroid: (**A**) Comparison between NTAT and benign tumours (*p* = 0.005), and between benign and malignant tumours (*p* = 0.004); (**B**) Comparison of the expression according to the histotype, with overexpression of FTA significantly different from FTC (*p* = 0.01) and NTAT (*p* = 0.004)–DGCR8 mutated case and the correspondent NTAT are in black; (**C**) Fold-change of DGCR8 mRNA expression using NTAT as a normalizer (=1) reveals that FV-PTC and FTC are the lesions that present the higher ratios for underexpression, in contrast with the other subtypes, being cPTC and FTA the lesions with a higher gain. * Kruskal–Wallis test statistical significance *p* < 0.05; ** Kruskal–Wallis test statistical significance *p* < 0.01.

The mRNA expression levels quantification of *DGCR8* were performed in 170 samples by qPCR. The expression of *DGCR8* was significantly different when comparing NTAT and benign tumours (Kruskal–Wallis test, *p < 0.01*) and between benign and malignant thyroid tumours (Kruskal–Wallis test, *p* < 0.01), Figure 1A. No differences were observed between NTAT and malignant tumours. For cPTC, FV-PTC, OV-PTC and PDTC no major differences were detected, Figure 1B. In contrast, for the remaining follicular cell-derived tumours, FTAs and FTC, significant alterations were identified. The *DGCR8* gene quantification in FTA cases revealed that this histotype presented a higher expression than all subgroups and were significantly higher than in NTAT (Kruskal–Wallis test, *p <* 0.01) and in malignant FTC cases (Kruskal–Wallis test, *p <* 0.05), Figure 1B. For optimal visualization of the changes between the different histotypes, a normalization was conducted with NTAT expression that was considered as basal expression and normalized to 1. Following normalization, the highest fold-changes were attributed to FTAs (1.75-fold change (fc)), followed by cPTC (1.35-fc), OV-PTC (1.42-fc) and PDTC (1.16-fc), Figure 1C. Contrarily to the latter, the follicular-patterned carcinomas FTC and FV-PTC, presented a reduction 0.84-fc and 0.92-fc, respectively, Figure 1C.

**Figure 2:**
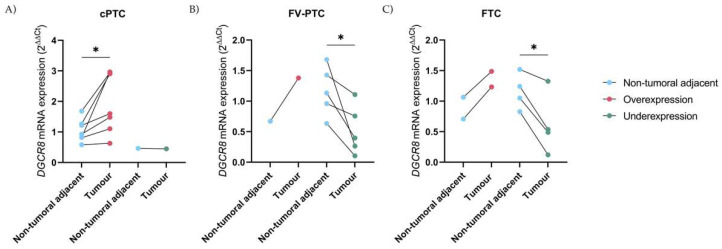
DGCR8 mRNA pairwise-matched tumour/NTAT analysis in cPTC(*n* = (8), FV-PTC (*n* = 6) and FTC (*n* = 6): (**A**) cPTC is characterized by overexpression and with significant difference in DGCR8 expression (*p* = 0.02); (**B**) In contrast to cPTC, in FV-PTC underexpression is more frequent and DGCR8 loss of expression is significant between tumours/NTAT (*p* = 0.04); (**C**) DGCR8 mRNA pairwise-matched tumour/NTAT expression in FTC cases. Four out of six cases presented a significant reduction in DGCR8 expression (*p* = 0.02). * Paired *t*-test statistical significance *p* < 0.05.

In 28 cases, the *DGCR8* gene expression between the tumour and its respective NTAT was available. The pairwise tumour/NTAT analyses revealed that in 87.5% (7 out of 8) of the cPTC cases, the main finding was overexpression of *DGCR8* in the tumours, with a statistically significant difference in expression (paired *t*-test, *p* < 0.05), Figure 2A. On the other hand, in FV-PTC cases and contrarily to the previous, underexpression was the most represented in 83.3% (5 out of 6), with statistically significant differences in expression (paired *t*-test, *p* < 0.05), Figure 2B.

The strong association of *DGCR8* downregulation in follicular-patterned carcinomas is also present in FTC, where underexpression is again present in most of the cases, 66.7% (4 out 6) and with significant differences (paired *t*-test, *p* < 0.05), Figure 2C. An interesting case in this series, was a multifocal PTC in a patient with two subtypes, a cPTC and a FV-PTC with Q61R *NRAS* mutation that in accordance with the previous findings, presented *DGCR8* overexpression and underexpression, respectively, in the different components. In the PDTC case with p.(E518K) mutation, the expression in tumour tissue was slightly lower than in NTAT, presenting similar patterns of expression with NTAT.

### 2.3. DGCR8 Immunoexpression in Thyroid Cancer

The expression of DGCR8 protein was performed in 99 FFPE samples and evaluated to create a score reflecting the staining intensity and extension, Table 2. Twenty-two cases (22.2%) were evaluated with a score of 0, however, only 4 cases lacked absolute expression for the DGCR8; the remaining 18 cases had less than 25% of extension. Regarding the other 77.8%, they were distributed throughout the additional score values as presented in Table 2.

**Figure 3:**
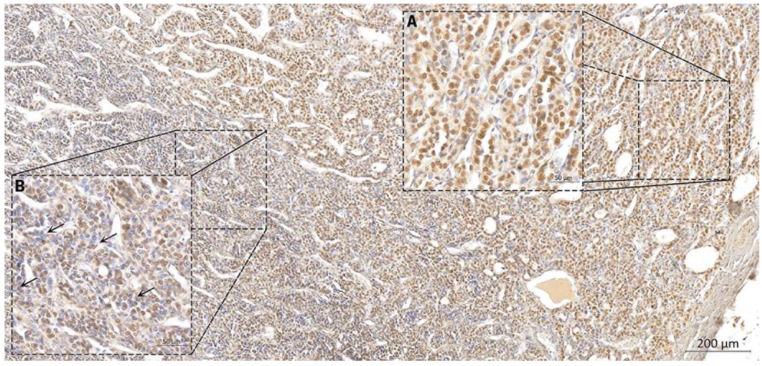
DGCR8 expression in the PDTC case with p.(E518K) mutation: (**A**) Overexpression of DGCR8 protein, with the highest expression in the series; (**B**) Some areas presented extensive loss of expression in some nuclei (black arrows) that putatively could be attributed due to LOH (as previously described in cases with p.(E518K) mutation).

As presented in Figure 3, the immunoexpression of DGCR8 was mainly found in the nucleus, as expected, and with an overall higher expression in lesion areas in comparison with NTAT. Stratification by histotype of the DGCR8 scores revealed that tendentially, higher median score patterns were associated with the PTC (with no statistical significance) (Table 2). The highest score of expression (9) was commonly detected in PTC (six cPTC and one FV-PTC) (7 out 15 cases with a score of 9, 46.7%) and followed by FTA (5 out 15 cases with score of 9, 33.3%). The PDTC were the most underrepresented histotype group (*n* = 4), and with three-quarters of the cases presenting low expression scores. Only one PDTC presented strong staining (score = 9) and it corresponded to the insular variant of PDTC case with DGCR8 p.(E518K) mutation, Figure 3A; this case presented the highest DGCR8 expression of all the evaluated samples. In some extensive areas of the mutated PDTC, it was noticeable that some nuclei presented loss of DGCR8 protein, Figure 3B (black arrows).

### 2.4. DGCR8 Expression, and Clinicopathological Associations

DGCR8 mRNA/protein expression was highly discordant and, overall, tendentially, presented a contrarywise behaviour, i.e., higher levels of *DGCR8* mRNA expression associated with lower protein scores. *DGCR8* mRNA expression was additionally compared to clinicopathological data but no significant associations were detected. The association of the mutation presence with clinicopathological data was not performed since the low number of events (only one *DGCR8* mutated case) precluded this analysis.

## 3. Discussion

The 22q region has been for a long time of major interest in thyroid lesions [22]. With the recent description of a *DGCR8* mutation (also located in 22q) in familial-MNG forms and sporadic thyroid carcinomas [2,7], we set to evaluate *DGCR8* as candidate gene in thyroid lesions. To date, the recurrent mutation *DGCR8* p.(E518K) [2,7] is the only mutation present in databases (TCGA) for thyroid lesions, and this was the major reason why we choose to perform only the characterization of *DGCR8* p.(E518K). We initiated by evaluating familial-associated MNG patients due to the previous reports- but we did not detect this alteration; the study comprised germline DNA evaluation of 12 index-cases, one for each family available. The next target were samples from sporadic cases, where only one case was found mutated. It corresponded to a PDTC with dominant insular pattern and with the recurrent missense mutation: c.1552G>A p.(E518K). Overall, for the PDTC histotype, the mutation frequency was 20.0%, a consequence of the reduced number of cases in the series, whereas, in the carcinomas, it was a rare event, 0.9% (1 out 109). It has been reported by Paulsson et al. [21] that mutations in *DGCR8* are recurrent in FTC, but in our series, we did not find any case mutated. In the previously reported (FV-PTC and FTC with p.(E518K)-mutation) it was also detected *NRAS* mutations concomitantly; this is in accordance with follicular-patterned tumours where *NRAS* is frequently found mutated [25]. It was advanced the *DGCR8* p.(E518K) mutation could influence the tumour progression or invasive behaviour without driving the tumour per se, since the tumours with this mutation are always described to bear additional genetic events [21]. In this case, no other molecular alterations were detected (*TERTp*, *BRAF* and *NRAS* hotspot mutations and *RET/PTC* and *PAX8/PPARg* rearrangements). This points that additional oncogenic events might be present or it may exist an exclusivity to non-classical events as previously presented by Chong et al. in *DICER1*-mutated thyroid carcinomas [26]. Possibly, *TP53* alterations that were already associated with miRNA biogenesis proteins in thyroid gland, are frequent in this histotype but were not evaluated; overall, this is the first report, so far, of a *DGCR8*-mutated PDTC.

The expression of *DGCR8* in benign tumours was significantly different from NTAT and malignant tumours. Although malignant tumours and NTAT are not significantly different, this may lay on the fact that the adjacent tissue of the tumour may already present altered expression. In the findings by Paulsson et al. [21], a downregulation of *DGCR8* gene expression in FTC in comparison to FTAs was reported; still, there were no data regarding the normal (or NTAT). We obtained similar results; however, we report that FTC change in expression is only significant when compared to FTAs but not NTAT. Aberrant expression of miRNA biosynthesis genes *DGCR8* and *DROSHA* are described to promote tumorigenesis as it results in aberrant miRNA expressional pattern that could be at play even at the level of tumour initiation, by downregulating tumour suppressor genes or overexpressing oncogenes [21,27]. These results suggest that overexpression of *DGCR8*, especially in FTA (the highest *DGCR8* expression), could be at play to force maintenance of “normal” thyroid differentiation, in particular, of the follicular differentiation and structure. On the other hand, follicular-patterned carcinomas of the thyroid (FTC and FV-PTC) displayed lower gene expression than NTAT, suggesting that not only mutations but also deregulation in expression takes part in tumorigenesis of thyroid follicular-patterned carcinomas as loss of differentiation occurs. Kim et al. [18] reported that the mRNA expression levels of *DGCR8* were found to be significantly lower in carcinomatous tissues as compared to the nonneoplastic tissues in a series of PTC; however, there are no data regard the variants present in this series. This is in accordance with our findings in human thyroid cell lines where papillary and anaplastic cell lines- TPC-1, T241 and 8505C, presented mRNA *DGCR8* expression lower than in human normal thyroid cell line- Nthy-ori-3-1. However, in our series only follicular-pattern carcinomas displayed lower mRNA *DGCR8* expression which can be justified by the genetic background of cell lines and its impact in *DGCR8* expression. When tumoral and NTAT matched-paired study was conducted, the follicular-patterned carcinomas (FTC and FV-PTC) presented more frequently a downregulation of *DGCR8* in comparison to their normal (NTAT) counterparts. The dichotomy cPTC/high *DGCR8* expression versus FV-PTC/low *DGCR8* expression was also present in the case of a patient with multifocal PTC and with two subtypes, a cPTC and a FV-PTC that presented overexpression and underexpression, respectively; this case illustrates the role of the deregulation of *DGCR8* mRNA in follicular-patterned carcinomas. A dependence of *DGCR8* in follicular-patterned carcinomas, already described by Paulsson et al. [21], is reported in this study with a statistical significative *DGCR8* mRNA underexpression in follicular-patterned carcinomas (FTC and FV-PTC) when compared to the normal counterpart. Beyond *DGCR8*, somatic *DICER1* mutations are reported in follicular-patterned lesions of thyroid (benign and malignant) which underlines the importance of the miRNA processing genes in follicular-patterned lesions [27,28,29].

In the PDTC with p.(E518K) mutation, the mRNA expression in tumour tissue was slightly lower than in NTAT with comparable expression to normal tissues but with a high protein expression as evaluated by IHC, in the tumour. Contrarily to what was observed, it was described that the p.(E518K) mutation could elevate *DGCR8* mRNA expression level, probably through interrupting miRNA binding [17,30] but we detected an expression comparable to the basal level.

DGCR8 immunoprofiling was performed by IHC and as expected, was mainly found in the nucleus. A tendency to more intense patterns were associated with PTC followed by FTA and FTC. PDTC cases presented low expression scores, except for PDTC case with *DGCR8* p.(E518K) mutation that presented strong staining (score of 9), the highest DGCR8 expression of all the evaluated samples. In areas of the mutated PDTC, it was noticeable that some nuclei presented loss of DGCR8 protein, possibly due to LOH, as reported in all *DGCR8* p.(E518K)-mutated cases so far. It would be interesting to determine if loss of the locus 22q is a second event in these cells that are losing the nuclear expression. Contrarily to DICER, where a positive correlation between expression at protein and RNA level is described [31], DGCR8 mRNA/protein expression was highly discordant and, in general, it behaved contrariwise; a perfect example was the PDTC with the mutation that had a high protein staining and low mRNA expression. This could be explained by the described autoregulatory feedback loop that when DROSHA and DGCR8 levels are elevated in the cell, the microprocessor cleaves and destabilizes the DGCR8 mRNA to reduce DGCR8 levels [32]. It is described that the knockdown of DROSHA leads to upregulation of DGCR8 expression at mRNA and protein levels, suggesting that not only alterations in DGCR8 but also alterations in other genes involved in miRNA biogenesis could alter the DGCR8 protein expression in thyroid lesions [18,33]. As this is the first study evaluating protein expression, further studies will help to clarify the mRNA and protein expression of DGCR8 in thyroid carcinomas.

The findings from this study strengthen the association between abnormal miRNA processing and the development and/or progression of thyroid cancer. As miRNAs are important to stabilize thyroid follicles and hormone production, it is perceivable that alterations in genes involved in miRNA biogenesis may have repercussions at follicular level, having a role in tumorigenesis of follicular-patterned tumours. In this study, these observations were particular evident in follicular-patterned carcinomas, suggesting that not only mutations but also alterations in DGCR8 mRNA/protein expression may be important in thyroid tumorigenesis. Succeeding *DICER1* alterations in the susceptibility of thyroid disease, we reaffirm *DGCR8* as another important player of the miRNA microprocessor complex team. It will now be an exciting endeavour to extend our series and clarify if E518K mutation drives alterations in miRNA and mRNA profiling.

## 4. Materials and Methods

### 4.1. Samples

The samples used in this study were collected at Centro Hospitalar e Universitário de São João (CHUSJ) and retrieved from the Department of Pathology of CHUSJ. The study was conducted in accordance with the Declaration of Helsinki, and the protocol was approved by the Ethical Committee of the CHUSJ (CES284-13), being an anonymized retrospective study, it was exempted from informed consent from patients in accordance with national ethical guidelines. All clinicopathological data were obtained from the anatomic pathology reports provided by the Department of Pathology from the CHUSJ. For the cases with available formalin-fixed paraffin-embedded (FFPE), tissues were re-evaluated, and histological diagnoses were reported according to the strict histomorphological criteria for current World Health Organization guidelines [34]. The following clinicopathological parameters were collected from the pathology reports: diagnosis, age at diagnosis, gender, tumour size, presence of tumour capsule, presence of capsular invasion, associated lesions, vascular invasion, lymph node metastasis, extrathyroidal invasion, presence of lymphocytic infiltrate, and other histological observations. According to the availability of adequate tissue, pathological report and/or clinical information, 226 samples from 209 patients were selected for the subsequent study; this included benign lesions, malignant lesions, and normal adjacent tissue samples. The included samples corresponded to: Normal tissue (NT) (*n* = 5); MNG (*n* = 15); follicular thyroid adenomas (FTA) (*n* = 86, 2 cases with non-tumoral adjacent tissue (NTAT) available); FTC (*n* = 22, 6 with NTAT); PTC (*n* = 80, 2 cases with NTAT); poorly differentiated thyroid carcinomas (PDTC) (*n* = 5, 1 case with NTAT available); and 2 cases of anaplastic thyroid carcinoma (ATC). Regarding the MNG, 12 cases were composed of germline DNA (blood) of probands from MNG with familial association. Part of this series was present in a biobank and was previously characterized for *BRAF*, *RAS*, and *TERT* promoter hotspot mutations, and *RET/PTC* and *PAX8-PPARg* rearrangements [24].

### 4.2. DGCR8 Amplification and Genotyping

*DGCR8* exon 6 was screened for mutations in DNA previously extracted from tumour tissues of all the samples described using PCR and Sanger sequencing [24]. The protocol used for PCR amplification was adapted from Rivera et al. [7] and the primers produced by IDT (IDT, Clinton, IA, USA). The following cycler conditions were used for PCR in MyCycler^TM^ Thermal Cycler (Bio-Rad, Hercules, CA, USA): 2 min at 95 °C, 35 cycles of: 20 s at 95 °C, 20 s at 62 °C and 20 s at 72 °C; and a final extension at 72 °C for 1 min. Following amplification, each amplicon was sequenced independently by using the corresponding forward and reverse primer at the following cycling conditions: 2 min at 95 °C, 40 cycles of: 15 s at 94 °C, 15 s at 55 °C, and 3 min at 60 °C; and a final extension at 60 °C for 10 min. The fragments were run in an ABI3130 Genetic Analyzer (Applied Biosystems, Foster City, CA, USA).

### 4.3. Quantitative PCR Analysis

Quantitative real-time PCR (qPCR) was performed when RNA of the frozen tissues was available and converted to cDNA using SuperScript™ IV cDNA Synthesis Kit according to the manufacturer’s instructions (Thermo Scientific, Waltham, MA, USA). For some of the samples, NTAT of the cases was included and analysed to create a pool of non-tumoral tissue. The qPCR evaluation was carried out in 170 samples, of which 5 were from NT, 28 corresponded to NTAT samples, 62 were FTA, 11 FTC, 61 PTC, and 3 PDTC. *DGCR8* mRNA expression was analysed using TaqMan PCR MasterMix (Applied Biosystems) and the amplification level was detected in a QuantStudio™ 5 Real-Time PCR System (Applied Biosystems), that was programmed to an initial step of 10 min at 95 °C, followed by 50 cycles of: 95 °C for 15 s and 60 °C for 1 min. Probes used for this analysis were: PrimeTime^®^ std qPCR Assay DGCR8 (Hs.PT.58.1414870 IDT) and the human TATA-binding protein (huTBP) gene (no. Hs.PT.39a.22214825, IDT) as endogenous control. Relative quantification of target genes was determined using the ΔΔ CT method, where similar amplification efficiencies between DGCR8 mRNA and huTBP were obtained, by Livak’s linear regression method [35].

### 4.4. Immunohistochemistry

Immunohistochemistry (IHC) was performed when FFPE tissues were available and it included 99 tumour sections, being: 30 FTA, 15 FTC, 50 PTC, and 4 PDTC. IHC was performed using Ultravision Quanto Detection System HRP (Thermo Scientific), according to the manufacturer’s instructions. Briefly, deparaffinized and rehydrated sections were subjected to heat-induced antigen retrieval for 45 min at 90 °C in 10 mM sodium citrate buffer (pH 6.0) (Thermo Scientific). Sections were incubated overnight at 4 °C in a humified chamber with anti-DGCR8 polyclonal antibody (PA5-40122, Invitrogen) at the optimized dilution of 1:250. The detection was performed with Polymer method detection system, HRP Polymer Quanto (Thermo Scientific) followed by 3,3′-diaminobenzidine (DAB) reaction and counterstained with Mayer’s hematoxylin. A normal thyroid sample was used as a positive control and the negative control consisted in the omission of the primary antibody. Slides were evaluated by an Endocrine Pathologist (S.C.) and an IHC score was established, which corresponded to the product of the intensity of expression (0 = negative; 1 = weak; 2 = intermediate; 3 = strong) with the tumour extent of protein expression (0: 0–25%; 1: 25–50%; 2: 50–75%; 3: 75–100%), 9 being the maximum score.

Slides were digitalized using a ZEISS axioscan 7 microscope slide scanner and pictures were treated in ZEISS 3.4. blue edition software (ZEISS, Oberkochen, Germany).

### 4.5. Statistical Methods

The statistical analysis was performed using GraphPad Prism version 9.0 (GraphPad Software, Prism, San Diego, CA, USA) and IBM SPSS version 25 (IBM, Armonk, NY, USA). Data were evaluated and tested for outliers’ determination and for normal gaussian distributions. Populations were compared with ANOVA; if failed gaussian distributions, with Kruskal–Wallis test. Comparison of tumours and NTAT was evaluated by paired *t*-test. For clinicopathological analysis association with the relative gene expression: gender, age, tumour size, diagnosis, histological characteristics, molecular status and DGCR8 expression were analysed using *t*-test and Mann–Whitney test. Results were considered statistically significant if *p* < 0.05.

## Figures and Tables

**Table 1:** Clinicopathological and molecular characterization of the series.

	Histological Subtypes							
Clinicopathological Variables *	FTA (*n* = 86)	MNG (*n* = 15)	FTC (*n* = 22)	cPTC (*n* = 44)	FV-PTC (*n* = 22)	OV-PTC (*n* = 14)	PDTC (*n* = 5)	ATC (*n* = 2)
Age (mean, y/o)	43.2	45.3	47.8	40.2	42.3	45	65.6	N.D.
Gender (female), *n* (%)	69 (80.2)	15 (100.0)	16 (72.7)	34 (77.3)	22 (100.0)	10 (71.4)	4 (66.7)	N.D.
Tumour size (mean, mm)	35	23	40	25	33	38	51	N.D.
Lymphocytic infiltrate, *n* (%)	20/67 (29.9)	N.D.	3/14 (21.4)	17/37 (45.9)	7/17 (41.2)	4/13 (30.8)	0/5 (0.0)	N.D.
Vascular invasion, *n* (%)	0/70 (0.0)	N.D.	8/17 (47.1)	19/37 (51.4)	5/18 (27.7)	6/13 (46.2)	2/3 (66.7)	N.D.
Lymph node metastasis, *n* (%)	0/1 (0.0)	N.D.	0/7 (0.0)	14/22 (63.6)	5/6 (83.3)	4/4 (100.0)	2/3 (66.7)	N.D.
Minimal extrathyroidal extension, *n* (%)	0/32 (0.0)	N.D.	1/14 (7.14)	15/37 (40.5)	5/18 (27.7)	7/12 (58.3)	2/5 (40.0)	1/1 (100.0)
Molecular characterization ^†^	**FTA**	**MNG**	**FTC**	**cPTC**	**FV-PTC**	**OV-PTC**	**PDTC**	**ATC**
*BRAF*, nm/nt (%)	0/23 (0.0)	0/2 (0.0)	0/18 (0.0)	18/42 (42.9)	4/20 (20.0)	4/13 (30.8)	0/4 (0.0)	0/1 (0.0)
*NRAS*, nm/nt (%)	4/86 (4.7)	0/2 (0.0)	4/21 (19.0)	2/42 (4.8)	3/21 (14.3)	2/12 (16.7)	0/4 (0.0)	0/1 (0.0)
*RET/PTC*, nm/nt (%)	0/74 (0.0)	0/2 (0.0)	1/13 (7.7)	5/36 (13.9)	0/20 (0.0)	2/14 (14.3)	0/4 (0.0)	N.D.
*PAX8/PPARg*, nm/nt (%)	3/76 (3.9)	0/2 (0.0)	3/16 (18.8)	0/41 (0.0)	1/21 (4.8)	0/14 (0.0)	0/4 (0.0)	N.D.
*TERTp*, nm/nt (%)	0/84 (0.0)	0/2 (0.0)	0/17 (0.0)	0/41 (0.0)	0/20 (0.0)	2/14 (14.3)	1/4 (25.0)	0/1 (0.0)
*DGCR8*, nm/nt (%)	0/86 (0.0)	0/15 (0.0)	0/22 (0.0)	0/44 (0.0)	0/22 (0.0)	0/14 (0.0)	1/5 (20.0)	0/2 (0.0)

Notes: * Not all samples had clinicopathological data available; ^†^ Not all samples were genotyped. nm: number of mutated samples; nt: number of total samples genotyped; N.D.: not determined.

**Table 2:** Score values of DGCR8 immunoexpression in the different histotypes.

	Score						
	0	1	2	3	4	6	9
**Histotype *n*, (%)**							
*FTA n* = 30	6 (20.0)	5 (16.7)	4 (13.3)	2 (6.7)	2 (6.7)	6 (20.0)	5 (16.7)
*FTC n* = 15	3 (20.0)	0 (0)	2 (13.3)	1 (6.7)	2 (13.3)	5 (33.3)	2 (13.3)
*PTC n* = 50	13 (26.0)	3 (6.0)	7 (14.0)	2 (4.0)	4 (8.0)	14 (28.0)	7 (14.0)
*PDTC n* = 4	0 (0)	1 (25.0)	2 (50.0)	0 (0)	0 (0)	0 (0)	1 (25.0)

## Data Availability

Not applicable.
